# Clinical application of a template‐guided automated planning routine

**DOI:** 10.1002/acm2.13837

**Published:** 2022-11-08

**Authors:** Matthew C. Schmidt, Christopher D. Abraham, Jiayi Huang, Clifford G. Robinson, Geoffrey Hugo, Nels C. Knutson, Baozhou Sun, Chipo Raranje, Erno Sajo, Piotr Zygmanski, Marian Jandel, Peter Szentivanyi, Jessica Hilliard, Jessica Hamilton, Francisco J. Reynoso

**Affiliations:** ^1^ Department of Radiation Oncology Washington University School of Medicine St. Louis Missouri USA; ^2^ Department of Physics University of Massachusetts Lowell Lowell Massachusetts USA; ^3^ Brigham and Women's/Dana Farber Cancer Institute/Harvard Medical School Boston Massachusetts USA; ^4^ Varian Medical Systems Palo Alto California USA

**Keywords:** automated planning, ESAPI, knowledge‐based planning, treatment planning

## Abstract

**Purpose:**

Determine the dosimetric quality and the planning time reduction when utilizing a template‐based automated planning application.

**Methods:**

A software application integrated through the treatment planning system application programing interface, QuickPlan, was developed to facilitate automated planning using configurable templates for contouring, knowledge‐based planning structure matching, field design, and algorithm settings. Validations are performed at various levels of the planning procedure and assist in the evaluation of readiness of the CT image, structure set, and plan layout for automated planning. QuickPlan is evaluated dosimetrically against 22 hippocampal‐avoidance whole brain radiotherapy patients. The required times to treatment plan generation are compared for the validations set as well as 10 prospective patients whose plans have been automated by QuickPlan.

**Results:**

The generations of 22 automated treatment plans are compared against a manual replanning using an identical process, resulting in dosimetric differences of minor clinical significance. The target dose to 2% volume and homogeneity index result in significantly decreased values for automated plans, whereas other dose metric evaluations are nonsignificant. The time to generate the treatment plans is reduced for all automated plans with a median difference of 9′ 50″ ± 4′ 33″.

**Conclusions:**

Template‐based automated planning allows for reduced treatment planning time with consistent optimization structure creation, treatment field creation, plan optimization, and dose calculation with similar dosimetric quality. This process has potential expansion to numerous disease sites.

## 1 INTRODUCTION

Treatment planning for inverse optimized external beam radiation therapy requires a complex trade‐off of parameterized dosimetric and deliverability objectives, and when combined with field design and modality selection, high levels of quality and standardization are difficult to achieve.^1^ Technologies and techniques have assisted in generating consistent quality plans by building relationships between the patient geometry and expected dosimetric output. Knowledge‐based planning (KBP) utilizes a library of prior plans to build a relationship among a patient contoured geometry, plan linear accelerator geometry, and expected dose distribution^2^, ^3^ or allows for the most similar contour geometry to drive fluence optimization.^4^, ^5^ More recently, these algorithms allow for three‐dimensional dose data to predict dosimetric outcomes relative to variations in patient geometry.^6^, ^7^, ^8^

As methods for correlating potential dose distribution to a cohort of input parameters (i.e., patient internal contoured geometry and gantry position parameters) have increased and vendor‐provided application programing interfaces (APIs) have facilitated interoperability between the treatment planning system (TPS) and custom software, automated treatment planning for external beam radiation therapy has become more feasible.^9^, ^10^, ^11^, ^12^ Variabilities in treatment planning goals and institution‐specific guidelines to radiotherapy have cultivated many site‐specific automated planning routines.^13^, ^14^, ^15^, ^16^, ^17^, ^18^, ^19^, ^20^, ^21^, ^22^ Templated approaches to plan automation have been introduced as a promising solution to meet clinician constraints and apply institutional practices.^23^, ^24^, ^25^

Whole brain radiation therapy (WBRT) is a common component of the treatment for brain metastases due to the decrease in overall tumor volume and improved control of new metastatic intracranial tumors, but patients undergoing WBRT have been shown to experience decline in cognitive function as well as general quality of life.^26^, ^27^ Hippocampal‐avoidance whole brain radiotherapy (HA‐WBRT), or avoidance of the hippocampal stem‐cell region, has demonstrated memory and cognitive function preservation and improved patient‐reported outcomes.^28^, ^29^ Irradiation of the whole brain while avoiding the hippocampus remains technically challenging even with modern delivery intensity‐modulated radiotherapy and volumetric modulated arc therapy (VMAT) due to the deep anatomic location of the hippocampus.^30^

A combination of vendor‐provided planning templates and KBP can provide enhanced standardization and efficiency to radiotherapy treatment planning. Although most automated planning routines require strict adherence to simulation or planning guidelines, this combined approach allows for the extension of automated planning routines to multiple treatment sites and techniques with additional templates. We have developed a template‐based automated planning application, QuickPlan, which utilizes commercial TPS templates and configurable parameters to automate the treatment planning process with HA‐WBRT as a clinical use case to present the validation and utility of generic automated planning.

## 2 MATERIALS AND METHODS

The purpose of QuickPlan is to simulate the treatment planning process in a single click while allowing the user the flexibility to make changes to dynamic parameters (i.e., treatment machine or prescribed dose) and providing integrity checks to ensure the structure set, field design, and optimization and dose calculation parameters are appropriate. The QuickPlan application is a .NET framework application developed in the C# programing language. The application interprets Extensible Markup Language‐formatted clinical templates (clinical protocols) from the TPS utilizing an open‐source class library solution, the Clinical Template Reader.^25^, ^31^ Our institutional templated planning solution for HA‐WBRT includes a 3‐arc VMAT delivery between 180.1 and 179.9 in alternating gantry travel direction. The collimator angles are 345, 15, and 90 degrees with the isocenter set at the center of the brain planning target volume (PTV) structure.

**FIGURE 1:**
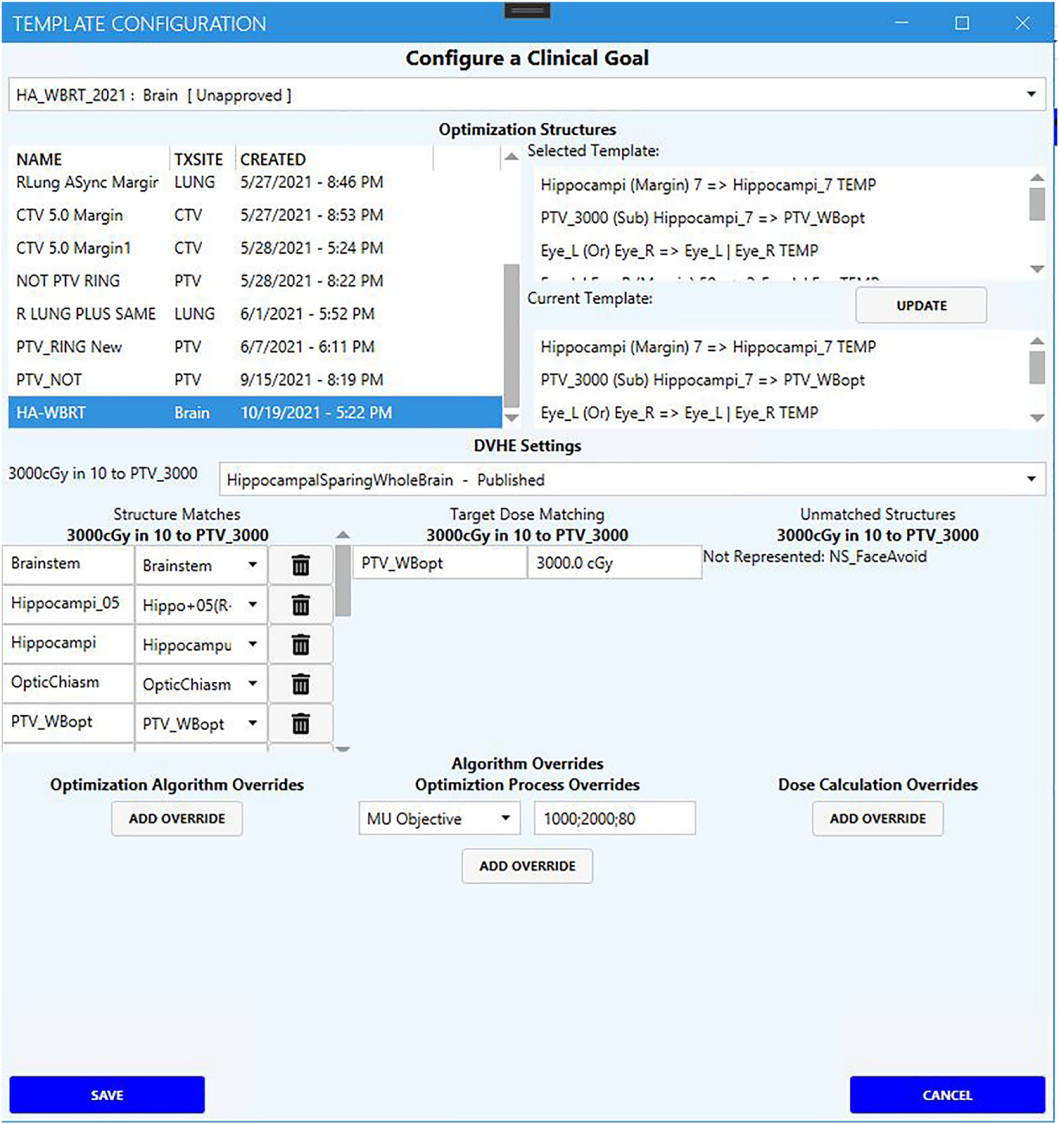
Configuration window for QuickPlan allows for the generation of automated planning templates, including automatically generated structures, knowledge‐based planning (KBP) structure matching, and algorithm setting overrides.

**FIGURE 2:**
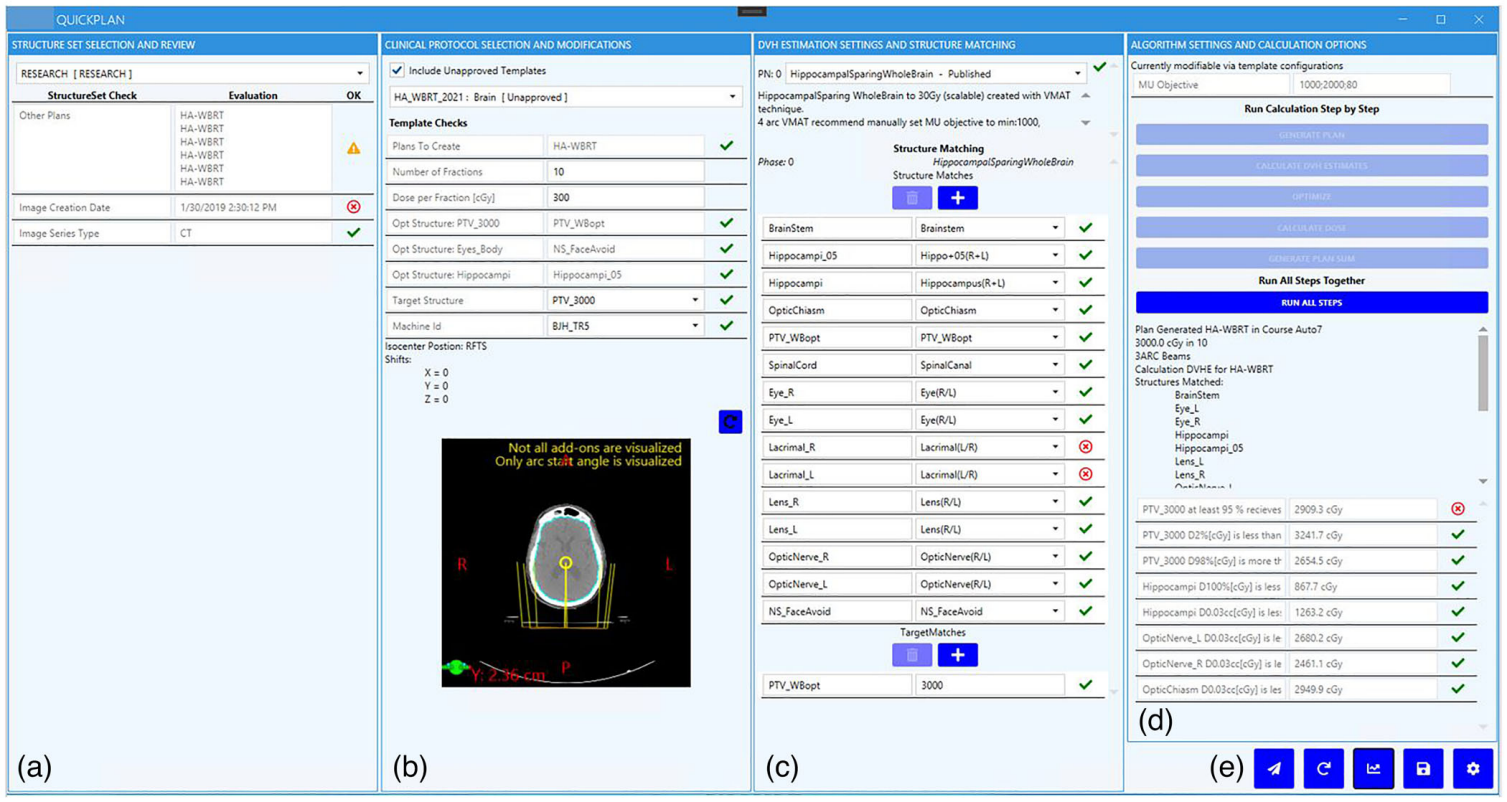
Planning user interface for QuickPlan includes panels for (a) structure set selection, (b) clinical protocol template selection, (c) knowledge‐based planning (KBP) structure matching and target matching, and (d) auto‐planning execution and plan review. The application contains a set of operations (e) for leaving messages for the developer, resetting the application, launching dose–volume histogram (DVH) visualization, saving, and launching the configuration window (left to right).

The Eclipse Scripting API (ESAPI, Version 15.6, Varian Medical Systems, Palo Alto, CA) performs the write‐enabled operations within the TPS, including the automation of planning organ‐at‐risk volume (PRV) generation and optimization with the commercial KBP optimization engine (RapidPlan, Eclipse v.15.6, Varian Medical Systems, Palo Alto, CA). The RapidPlan model utilized in HA‐WRBT planning is available from the Varian Medical Affairs website (http://medicalaffairs.varian.com/wholebrain‐hippocampalsparing‐vmat1). A configuration page within QuickPlan allows for the template‐specific automation steps not available within the clinical protocol templates, including automated optimization structure and PRV creation, KBP optimization structure and target matching, and modifications to algorithm parameters for the specified treatment site (Figure 1). The application user interface (UI) is partitioned into four planning sub‐modules with separated responsibilities (Figure 2).

On application launch, the structure set selected in the TPS is automatically selected as the primary planning structure set (Figure 2a). Once the user selects the clinical protocol, QuickPlan will interpret the template using the Clinical Template Reader open‐source library to display the prescription, target, isocenter location, and machine ID, and the configuration will generate the PRVs based on a predefined set of the Boolean operation instructions (Figure 2b). The geometry and isocenter placement are visualized in the second panel for user review. The KBP model is selected automatically from the configuration template with specified structure matching and target dose level matching (Figure 2c). Finally, the user triggers the automated planning and receives status updates, visualizations, and dose metric evaluations (Figure 2d). The user has a panel of actions that allow for the following operations: (1) send a message to the developer if issue is found in planning, (2) reset the application and start again, (3) open a review space for dose–volume histogram estimates and dose–volume histogram (DVH) review, (4) save modifications in the TPS, and (5) open the configuration page (Figure 2e, left to right).

**FIGURE 3:**
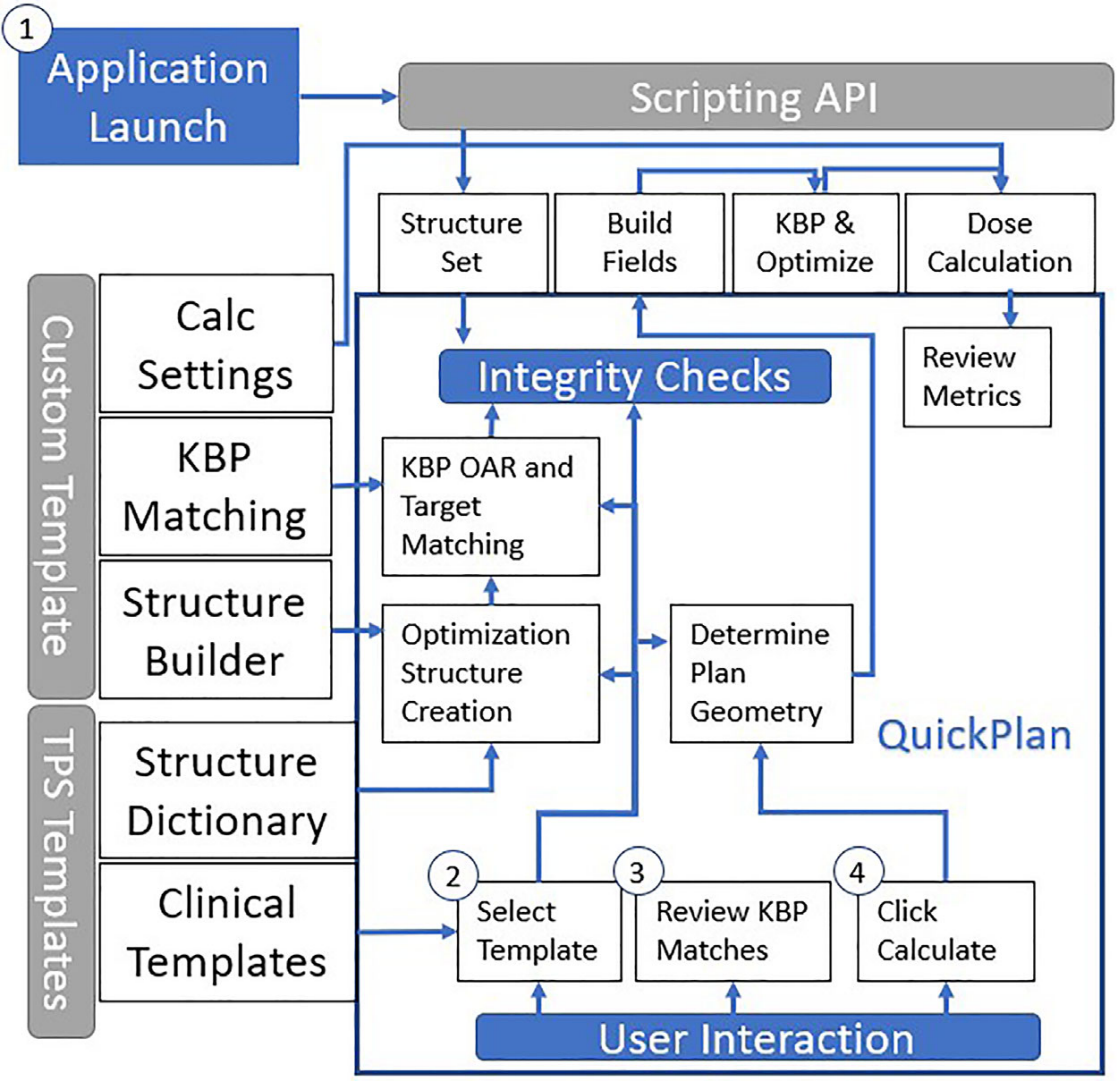
Workflow for QuickPlan, including (1) the launching of the application, (2) the selection of the clinical template, (3) the review of the knowledge‐based planning (KBP) structure matching, and (4) the generation of the plan along with associated templates to provide guidance

QuickPlan interacts with multiple components of the TPS and requires minor interaction from the user (Figure 3). The scripting API provides contours for field fitting, optimization structure creation, and optimization (Step 1, Figure 3) and methods for building the treatment beams, applying the KBP model, optimizing, and calculating dose (Step 4, Figure 3). The field design, prescription, target volumes, and default treatment machine are available through directly reading and translating the TPS planning template files. Configurable custom templates within the QuickPlan application control the creation of structures for optimization through a custom “structure‐builder” class library—a compiled set of methods that build contours from the Boolean operations and margins from already existing contours. An interpretation layer for the KBP model assists in the selection of planning structures to match with the KBP model structures and custom algorithm settings for optimization and dose calculation. The KBP model interpretation layer may be useful when structures within the model have a one‐to‐many relationship with contoured structures or structure names do not match exactly. Finally, due to a nonconformance of structure names between the KBP model and the institution, an XML‐formatted TPS‐provided structure dictionary has been repurposed from the Visual Scripting application (Varian Medical Systems, Palo Alto, CA) to assist in the finding of structures within the structure set if the structure identifiers do not match. TPS and custom templates are applied collectively to automate the optimization structure creation process, the isocenter placement, field design, and KBP structure matching as the user selects a TPS planning template (Step 2, Figure 3). However, the user can manually modify the structure matches at runtime (Step 3, Figure 3).

Each panel to the UI has integrity checks that are tracked and logged throughout the life of the application (Table 1). Some planning and dosimetric validations assist the user in clinical decision support—for example, the date of CT scan creation could notify the user that an image is selected from a prior course of treatment. Other validations are tracked to determine if the user has modified the parameter prior to planning to be used as a feedback mechanism for application improvement and future automated clinical decision support.

**TABLE 1:** Validations performed at various planning process steps to assist user in clinical decision support or track parameter changes for future improvement

Panel	Check	Description	Possible states
Structure set selection and review	Other plans	If structure set is used in any other plans, it could indicate old structure set	Pass or warning
	Image creation date	Check if image is more than 14 days old	Pass or flag
	Image series type	Check to ensure planning image is CT	Pass or flag
Clinical protocol selection and modification	Plan to create	Evaluates clinical protocol interpretation	Pass or flag
	Number of fractions	Interpreted from clinical protocol	Tracked for changes
	Dose per fraction	Interpreted from clinical protocol	Tracked for changes
	Optimization structure creation	Successful generation of PRV from configuration template	Pass or flag
	Target^a^	Successful matching of clinical protocol template target to planning structure	Pass or flag and tracked for changes
	Treatment machine	Interpreted from clinical protocol	Tracked for changes
DVH estimation and structure matching	Planning structure name (left)^a^	Successful matching of planning structure to configuration template	Pass or flag and tracked for changes
	KBP structure match (right)	KBP model structure from configuration template	Tracked for changes
	Target name (left)^a^	Successful matching of configuration template target to planning structure	Tracked for changes
	Target dose (right)	Target dose level(s) from configuration template	Tracked for changes
Algorithm settings and calculation options	Clinical goal	Evaluated clinical goal after dose calculation	Pass of flag

Abbreviations: DVH, dose–volume histogram; KBP, knowledge‐based planning; PRV, planning organ‐at‐risk volume.

^a^Structures Ids additionally matched to structure dictionary.

QuickPlan validation was performed by the automated planning of HA‐WBRT VMAT for 22 retrospective patients. These validation plans were also manually replanned with a certified dosimetrist with significant HA‐WBRT planning experience mimicking the steps executed by the QuickPlan's internal algorithm. Dose quality metrics from NRG‐CC001 are evaluated between the manual and automated plans and average DVHs, normalized to the clinical trial requested prescription dose to 95% of the target volume, and are visualized between each planning technique.^29^ Additionally, the PTV homogeneity index using the difference between the dose at 5% and 95% volumes normalized to the prescription dose are added to give clinical context to plan quality.^32^ Statistical significance is determined at a *p*‐value of 0.05 determined by a Wilcoxon signed‐rank test.

A technical planning time (TPT) evaluates the time from the plan's initial creation to the end of the optimization, including the generation of derived structures used in optimization. This time was extrapolated from calculation logs in the TPS and only captures active treatment planning time. This time range selected due to the availability of precise end points in the calculation logs of the TPS. TPT is evaluated in between automated plan and manual replan of retrospective patients. After this validation, 10 prospective patients were planned for treatment using the QuickPlan with planning time reported. For both QuickPlan retrospective and automated plans, the time to execute (TTE) is extracted from the application logs to show the amount of time spent in the application prior to plan creation. TTE is prior to the start of TPT and represents the amount of time passed between launching the application and executing the plan creation.

## 3 RESULTS

**FIGURE 4:**
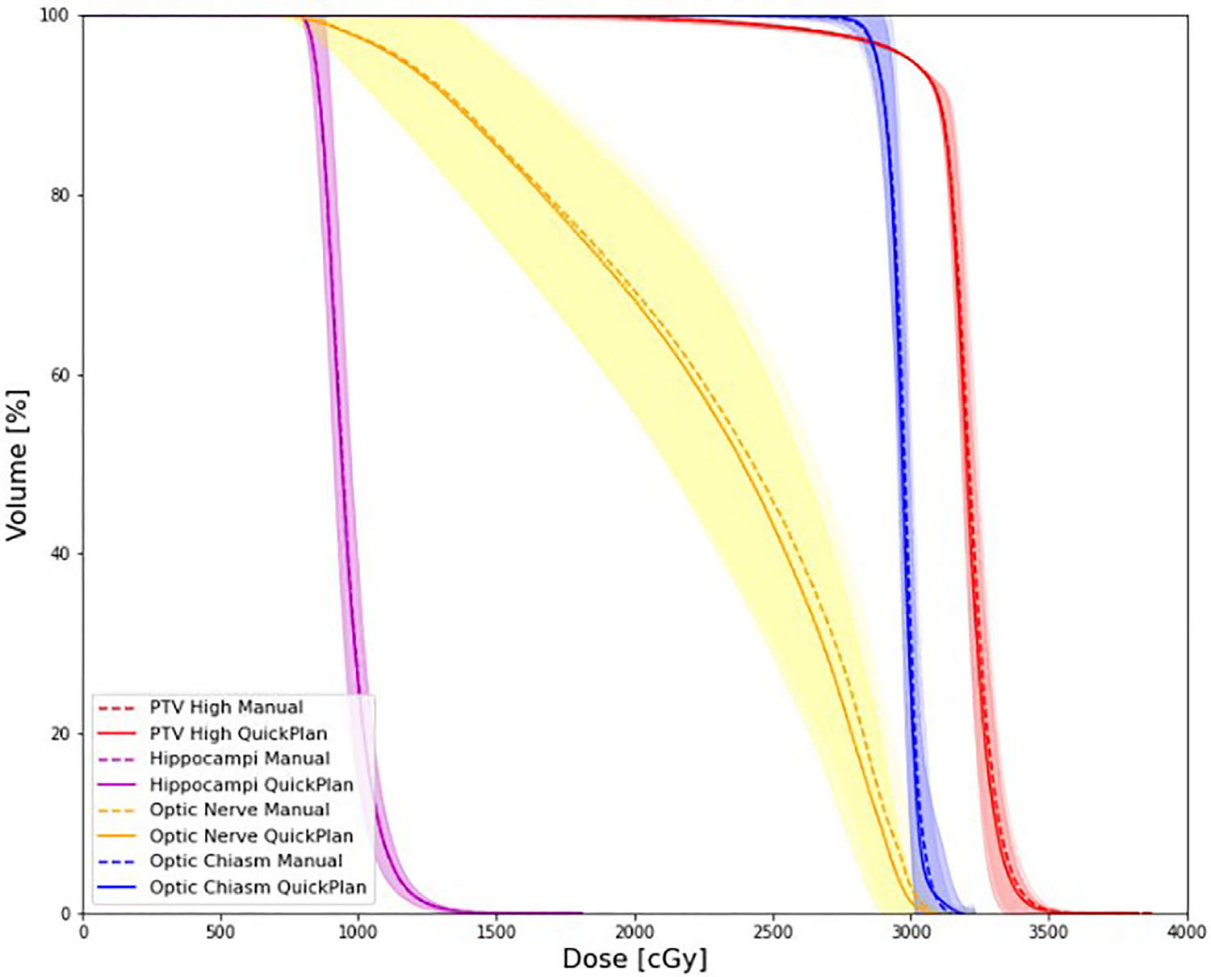
Dose–volume histogram (DVH) of clinical structures evaluated between manually replanned and QuickPlan automated validation cases. Shaded regions denote the standard deviation at each dose bin within the DVH.

The mean DVHs for structures of consideration are shown for all 22 validation cases with automated plans generated by the QuickPlan application and manual plans replanned utilizing the same procedure embedded into the QuickPlan configuration (Figure 4). Hippocampi and optic nerve structures represent the combination of their bilateral contours. Shaded regions denote the standard deviation at each dose bin within the DVH.

Dosimetric goals compared across the automated and manual replan patients are shown in Table 2. The difference in these metrics is represented as the QuickPlan planned dose metric value subtracted from the manual replan. Only two metrics show statistical significance, the dose to 2% of the target volume and the homogeneity index, but these metrics, along with all others, are considered equivalent in their clinical significance.

**TABLE 2:** Manual and automated treatment plan dose metric evaluations for planning target volume (PTV) and critical structures

Structure	Metric	Goal	Manual (Gy) (*σ*)	Auto (Gy) (*σ*)	Difference (Gy) (*p*)
PTV_3000	*D*2% (Gy)	≤37.5	34.00 (0.43)	33.82 (0.36)	**0.14 (0.001)**
	*D*98% (Gy)	≥25	26.93 (0.82)	26.94 (0.80)	−0.01 (0.610)
	*V*30 Gy (%)	≥95	94.99 (0.01)	94.99 (0.01)	0.00 (0.505)^a^
	PTV HI	N/A	4.42 (0.51)	4.25 (0.41)	**0.17 (0.001)**
Hippocampi	*D*100% (Gy)	≤9	8.32 (0.32)	8.33 (0.33)	−0.01 (0.999)
	*D*0.03 cm^3^ (Gy)	≤16	12.75 (0.99)	12.76 (0.67)	−0.39 (0.799)
Optic nerve	*D*0.03 cm^3^ (Gy)	≤30	29.17 (0.99)	28.97 (1.21)	0.20 (0.165)
Optic chiasm	*D*0.03 cm^3^ (Gy)	≤30	30.37 (0.36)	30.34 (0.42)	0.03 (0.234)

*Note*: Statistical significance is given in boldface.

^a^Dose–volume histograms prescribed dose normalized to 95% of target volume.

**FIGURE 5:**
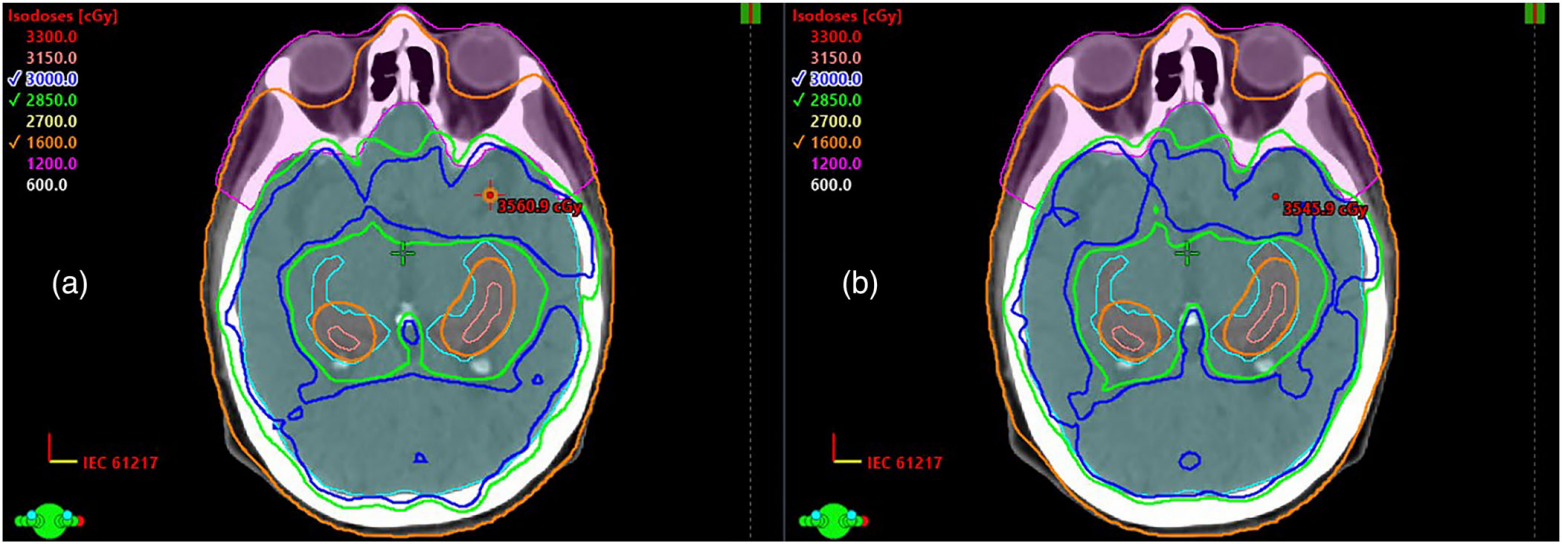
Isodose distribution for hippocampal‐avoidance whole brain radiotherapy (HA‐WBRT) (a) automated plan and (b) manual plan overlay with planning target volume (PTV) (cyan), hippocampi (pink), and NS_FaceAvoid (magenta)

Isodose line comparison is shown between a single automated plan and manual plan (Figure 5). The PTV and bilateral hippocampi are displayed in cyan and pink, respectively. The NS_FaceAvoid structure, shown in magenta, is generated by QuickPlan's structure‐builder library along with a 5 mm ring (NS_Ring_05), a PRV margin on the hippocampi (Hippocampi_05), and a target for optimization (PTV_WBopt), which are not shown. More details on the optimization PTV can be found in the clinical description of the published KBP model.^33^ The 1600 cGy isodose line is displayed as the sparing goal for the hippocampi, and the prescription and 95% isodose line are visualized for target coverage.

**FIGURE 6:**
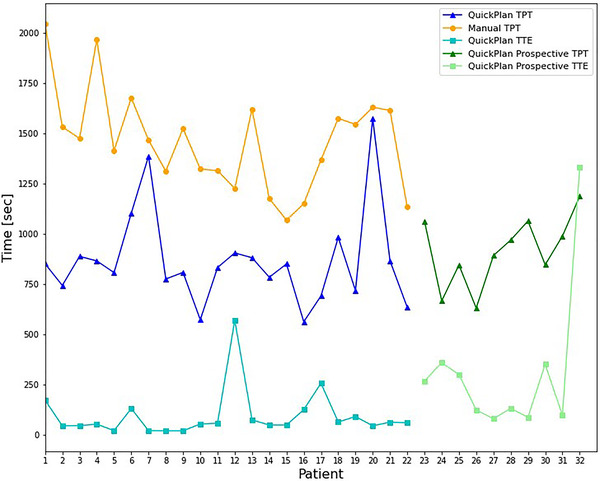
Technical planning time (TPT) and time to execute (TTE) evaluations for automated and manual treatment planning techniques, including prospective cases planned in clinical practice with QuickPlan

TPT evaluation showed consistently lower calculation times for QuickPlan automated plans when compared to manual replans with the same procedures (Figure 6). The TPT for manual plans ranged from 17′ 50″ to 34′ 7″, whereas the automated plans ranged from 9′ 23″ to 26′ 16″. The median planning time was 24′ 32″ ± 4′ 5″ and 14′ 2″ ± 3′ 51″ for manual and automated plans, respectively. For prospective patient plans generated with QuickPlan, the median planning time is 15′ 31″ ± 2′ 47″ across 10 plans. TTE ranged from 20″ to 9′ 31″ ± 56″ in retrospective QuickPlan validation and from 1′ 20″ to 20′ 11″ ± 5′ 55″ in prospective QuickPlan planning. The increase in the median planning between retrospective and prospective QuickPlan use from 1′ 57″ to 2′ 40″ is likely due to the increased scrutiny in validation parameters for clinically deliverable plans and an unfamiliarity with the tool to new dosimetry users. Removing the maximum times for each TTE dataset, the only times greater than 2 standard deviations from the median, the median TTE is 54″ and 2′ 12″ for retrospective and prospective automated planning, respectively. Distractions between launching the application and executing the plan creation contribute high TTE outliers to both auto‐planned datasets.

## 4 DISCUSSION

QuickPlan as a platform allows for the combination of TPS planning templates with numerous customizable templates to drive the user through the planning process in an efficient and reproducible manner. An internal structure‐builder library methodically generates PRVs, dose control, and other optimization structures. Once the user selects the planning template, the treatment beam generation, KBP DVH estimation, optimization, dose calculation, and dosimetric objective evaluation are available with a single click of a button. Homogenization of KBP model structures and planning structures, which can be difficult if the KBP model is not built in‐house, and more intelligent target detection and prescription determination could allow the user less time in review or to even forgo the UI altogether.

HA‐WBRT provides a convenient initial clinical planning site for an automated planning routine due to the minor geometric variabilities among patients, clear clinical goals, and contouring guidelines.^13^, ^20^ Additionally, our institution's lack of experience with HA‐WBRT allowed for the development of consistent planning procedures that could be mirrored within a software routine, and the automated plan creation streamlined the institution's procedure validation. The initial validation of the QuickPlan shows template‐based automated planning yields clinically similar plans dosimetrically while requiring considerably less time. As Figure 6 shows, automated treatment planning has lower TPT in all validation cases. TPT for automated plans benefit from the field generation and optimization contour creation as the use of the clinical TPS UI to accomplish these tasks require even a familiar user to utilize multiple workspaces (Contouring and External Beam Planning) and numerous clicks. As our institutional TPS is cloud‐based, variation in the optimization portion of the TPT is induced by the differences in relative computation speed due to network traffic throughout the day and challenges to the optimizer in reaching an acceptable objective function value. The TPT deduced from the calculation logs in the TPS only captures active treatment planning time and yields a limited scope of the full efficiency gains a template‐based planning automation application can produce. The ability to produce clinically acceptable plans that are equivalent to manually produced plans can produce significant passive time savings where a planner may be able to work on other plans, whereas the automated routine generates a plan.

Additional disease sites to be added to QuickPlan are currently undergoing investigation. The process for implementing new treatment sites is as follows:

1. Determine contoured targets and structures. Generate structure template for consistent naming.
2. Generate clinical protocol with treatment field definitions, default treatment machine and targets, and clinical dosimetric goals.
3. Identify optimization structures and generate a templated set of instructions for the derivation of these contours and assign template to clinical protocol in QuickPlan configuration.
4. Identify any algorithm setting deviations from the defaults and assign to clinical protocol template in the QuickPlan configuration.
5. Generate KBP model structure mapping in QuickPlan configuration.

Challenges exist in the implementations of new disease sites that are staged for future works. This includes the optimization of collimator rotation angle to optimize the PTV geometric coverage in treatment sites like chest wall or pelvis. Future development for QuickPlan also includes searching for additional targets from available physician's intent orders or prescriptions as opposed to relying on static templates as additional targets or nodal volumes are difficult to predict in template form.

## 5 CONCLUSION

Automated planning software with configurable contouring, field design, and KBP templates and UI‐driven real‐time validation allow for the generation of user‐verified treatment plans of similar dosimetric quality while reducing required planning time. A consistent clinical protocol that includes clear clinical goals and contouring guidelines is required for template‐based automation to succeed.

## AUTHOR CONTRIBUTIONS

Matthew C. Schmidt, Francisco J. Reynoso, and Geoffrey Hugo contributed the initial ideas that lead to the implementation of the software application mentioned in the project. Matthew C. Schmidt and Peter Szentivanyi programed the software. Christopher D. Abraham, Jiayi Huang, Clifford G. Robinson, Nels C. Knutson, and Baozhou Sun contributed to the clinical knowledge required to implement the tool safely in clinical practice. Chipo Raranje, Jessica Hilliard, and Jessica Hamilton assisted in the validation of the tool against current clinical standards. Matthew C. Schmidt wrote the manuscript. Erno Sajo, Piotr Zygmanski, and Marian Jandel assisted in guiding the manuscript for completeness, and all authors contributed to the review and revision of the manuscript.

## CONFLICT OF INTEREST

Matthew C. Schmidt reports consulting fees and honoraria with Varian Medical systems, inc and Lifeline Software, LLC unrelated to this work. Geoffrey Hugo reports grants from the American Heart Association, Varian Medical Systems, and Viewray, Inc. and personal fees from Varian Medical Systems outside the submitted work. In addition, Dr. Hugo has patents pending licensed to Varian Medical Systems outside of the submitted work. Francisco J. Reynoso reports honoraria for teaching profressional services with Varian Medical Systems unrelated to this work. Clifford G. Robinson has received grants from Varian Medical Systems, Inc and Elekta AB and consulting and speaking honoraria from Varian Medical Systems, Inc and Viewray Technologies, Inc unrelated to this work. Nels C Knutson has received research grants from Varian Medical Systems, Inc and consulting and speaking honoraria from Varian Medical Systems, Inc unrelated to this work. All other authors report no conflicts of interest.

## Data Availability

The data that support the findings of this study are available from the corresponding author upon reasonable request.
